# COVID-19 in Elderly, Immunocompromised or Diabetic Patients—From Immune Monitoring to Clinical Management in the Hospital

**DOI:** 10.3390/v14040746

**Published:** 2022-04-01

**Authors:** Korbinian Wünsch, Olympia E. Anastasiou, Mira Alt, Leonie Brochhagen, Maxim Cherneha, Laura Thümmler, Lukas van Baal, Rabea J. Madel, Monika Lindemann, Christian Taube, Oliver Witzke, Hana Rohn, Adalbert Krawczyk, Sarah Jansen

**Affiliations:** 1 West German Centre of Infectious Diseases, Department of Infectious Diseases, University Hospital Essen, University Duisburg-Essen, 45147 Essen, Germany; korbinian.wuensch@uk-essen.de (K.W.); mira.alt@uk-essen.de (M.A.); leonie.brochhagen@uk-essen.de (L.B.); maxim.cherneha@uk-essen.de (M.C.); laura.thuemmler@stud.uni-due.de (L.T.); rabea.madel@uk-essen.de (R.J.M.); oliver.witzke@uk-essen.de (O.W.); adalbert.krawczyk@uk-essen.de (A.K.); 2 Institute for Virology, University Hospital Essen, University Duisburg-Essen, 45147 Essen, Germany; olympiaevdoxia.anastasiou@uk-essen.de; 3 Institute for Transfusion Medicine, University Hospital Essen, University Duisburg-Essen, 45147 Essen, Germany; monika.lindemann@uk-essen.de; 4 Department of Endocrinology, Diabetes and Metabolism and Division of Laboratory Research, University Hospital Essen, University Duisburg-Essen, 45147 Essen, Germany; lukas.van-baal@uk-essen.de; 5 Department of Pneumology, University Medicine Essen—Ruhrlandklinik, University Duisburg-Essen, 45147 Essen, Germany; christian.taube@rlk.uk-essen.de

**Keywords:** SARS-CoV-2, COVID-19, comorbidities, immunocompromised, diabetes mellitus, elderly patients

## Abstract

The novel, highly transmissible severe acute respiratory syndrome coronavirus 2 (SARS-CoV-2) has triggered a pandemic of acute respiratory illness worldwide and remains a huge threat to the healthcare system’s capacity to respond to COVID-19. Elderly and immunocompromised patients are at increased risk for a severe course of COVID-19. These high-risk groups have been identified as developing diminished humoral and cellular immune responses. Notably, SARS-CoV-2 RNA remains detectable in nasopharyngeal swabs of these patients for a prolonged period of time. These factors complicate the clinical management of these vulnerable patient groups. To date, there are no well-defined guidelines for an appropriate duration of isolation for elderly and immunocompromised patients, especially in hospitals or nursing homes. The aim of the present study was to characterize at-risk patient cohorts capable of producing a replication-competent virus over an extended period after symptomatic COVID-19, and to investigate the humoral and cellular immune responses and infectivity to provide a better basis for future clinical management. In our cohort, the rate of positive viral cultures and the sensitivity of SARS-CoV-2 antigen tests correlated with higher viral loads. Elderly patients and patients with diabetes mellitus had adequate cellular and humoral immune responses to SARS-CoV-2 infection, while immunocompromised patients had reduced humoral and cellular immune responses. Our patient cohort was hospitalized for longer compared with previously published cohorts. Longer hospitalization was associated with a high number of nosocomial infections, representing a potential hazard for additional complications to patients. Most importantly, regardless of positive SARS-CoV-2 RNA detection, no virus was culturable beyond a cycle threshold (ct) value of 33 in the majority of samples. Our data clearly indicate that elderly and diabetic patients develop a robust immune response to SARS-CoV-2 and may be safely de-isolated at a ct value of more than 35.

## 1. Introduction

Coronavirus disease (COVID-19) is an ongoing global threat with more than 200 million confirmed cases and over 5 million deaths worldwide [1]. In December 2020, the number of COVID-19 patients rose dramatically to occupy 82% of the intensive care (ICU) beds in Germany [2]. The alpha variant was predominant in Germany until May 2021 [3]. One year later, global health systems continue to face a shortage of medical resources [4]. Despite the available and efficient vaccines, COVID-19 remains a major challenge for healthcare systems, requiring a massive number of medical resources. As a consequence of the spread of the even more contagious Omicron variant, the number of SARS-CoV-2 infections is rising again, and the occupancy of ICU beds is reaching 88%. The majority of patients requiring intensive care are over 60 years of age (more than 60%) [5].

Given the current evolution of the pandemic, it is more urgent than ever to provide appropriate treatment to all hospitalized COVID-19 patients. In Germany, isolation after recovery is recommended for at least 14 days [6,7]. The clinical management of COVID-19 patients requiring outpatient care or mobile care services after the acute phase of the disease, such as elderly patients or patients with comorbidities, remains challenging. End-of-isolation measures for these patients requires a negative SARS-CoV-2 polymerase chain reaction (PCR) test result or, at least, a cycle threshold (ct) value over 30. However, although most of these patients recover from infection, many remain SARS-CoV-2 PCR-positive for an extended period. Thus, they continue to receive inpatient treatment to minimize the risk of SARS-CoV-2 transmission when released to outpatient care facilities. Age over 60 years has been identified as a major risk factor for severe COVID-19 [8]. Further known comorbidities associated with severe COVID-19 include immunocompromisation [9] and diabetes mellitus (DM) [10,11,12]. Of note, patients with severe COVID-19 may exhibit infectious viruses and often have high ct values for a longer period than patients with a moderate COVID-19 infection [13], thus further limiting the capacity of the health care system. This results in a high number of patients for whom there are no clearly defined guidelines for the duration of isolation.

Viral excretion kinetics are less well-studied in very elderly patients with pre-existing conditions. To assess the potential risk of infection in recovered but still SARS-CoV-2 RNA-positive “at-risk” patients, we examined the cellular and humoral immune responses, SARS-CoV-2 RNA levels, and viral load of 79 COVID-19 patients. All patients were hospitalized at the Department of Infectious Diseases, University Hospital Essen, Germany, between December 2020 and April 2021.

## 2. Materials and Methods

### 2.1. Study Population

This prospective study included 79 patients with a polymerase chain reaction (PCR)-confirmed SARS-CoV-2 infection. Patients were hospitalized at the Department of Infectious Diseases, University Hospital Essen, Germany, between December 2020 and April 2021. Inclusion criteria were a PCR-confirmed SARS-CoV-2 infection, age of 60 years or older, immunocompromisation or diabetes mellitus, and ongoing hospitalization. A total of 56 patients manifested with severe COVID-19 and 23 patients had moderate COVID-19 at the time of enrollment. SARS-CoV-2 RNA was measured from nasopharyngeal swabs taken at the time of hospitalization and approximately 10 to 15 days after the first positive PCR test. A follow-up SARS-CoV-2 PCR was performed approximatel 7 days later, if the second SARS-CoV-2 PCR was positive and the patients were still hospitalized. Patients were classified into the following groups according to the predominant risk factors for severe COVID-19: (i) immunocompromised patients (regardless of age; mean age 63.3 years with a standard deviation (SD) of ±17.7 days), (ii) patients with diabetes mellitus (regardless of age; mean age 69.6 with an SD of ±9.9 years), and (iii) elderly patients over 60 years of age who were neither immunocompromised nor had diabetes mellitus (referred to as elderly patients in the following; mean age 73.4 with an SD of ±10.1 years). Detailed patient characteristics are shown in Table 1. Notably, none of the patients were vaccinated against SARS-CoV-2 prior infection. Regarding the length of hospital stay, one patient was excluded from the analysis. This patient was hospitalized for more than 170 days and only a fraction of that time was due to COVID-19. The study was approved by the local ethics committee (approval numbers 20-9512-BO and 20-9225-BO) and was carried out according to the ethical standards of the Declaration of Helsinki of 1964 and its subsequent amendments or comparable ethical standards.

### 2.2. Cells and Virus

Vero E6 cells (American Type Culture Collection (ATCC), CRL-1586, Rockville, MD, USA) were cultured in Dulbecco’s modified Eagle’s medium (DMEM Life Technologies Gibco, Darmstadt, Germany) supplemented with 10% (*v*/*v*) fetal calf serum (FCS), penicillin (100 IU/mL), and streptomycin (100 µg/mL) at 37 °C in an atmosphere of 5% CO_2_. Nasopharyngeal swab specimens were titrated on Vero E6 cells within 48 h to detect infectious virus. The medium was additionally supplemented with Ciprofloxacin (10 mg/mL), and Amphotericin B (2.5 mg/mL) to avoid contamination. After 72 h, cell cultures were analyzed for cytopathic effects (CPE) by transmitted light microscopy (Carl Zeiss AG, Oberkochen, Germany). Supernatants from infected cell cultures were harvested after 7 days of infection, cleared from any cell debris by centrifugation, and stored at −80 °C. A SARS-CoV-2 clinical isolate from 04/2020 with the D614G mutation was used for the virus neutralization assays.

### 2.3. SARS-CoV-2 Specific Antibody Detection

SARS-CoV-2 immunoglobulin (Ig)G against the spike glycoprotein was detected using an anti-SARS-CoV-2 IgG chemiluminescence enzyme immunoassay (CLIA) (LIAISON^®^ SARS-CoV-2 TrimericS IgG assay, Diasorin, Saluggia, Italy) according to the manufacturer’s instructions. A result of ≥13.0 arbitrary Units per milliliter (AU/mL) was considered positive and a score of <13 AU/mL was considered negative. The upper limit of quantification was 800 AU/mL. The conversion factor for the results in AU/mL to Binding Antibody Units (BAU)/mL, which correlates with the WHO International Standard for COVID-19, was 2.6 [14,15].

### 2.4. Virus Neutralization Assay

The neutralizing capacity of serum samples was determined by a standard end-point dilution assay, as previously described [16]. In brief, two-fold serial dilutions of patient sera (1:20 to 1:2560) were pre-incubated with 100 TCID_50_/50µL SARS-CoV-2 for one hour at 37 °C. The mixtures were added to confluent Vero E6 cells cultured on 96-well microtiter plates supplemented with 10% FCS, penicillin (100 IU/mL) and streptomycin (100 µg/mL). To detect cytopathic effects (CPEs) via light microscopy, the cell cultures were stained with crystal violet (Roth, Karlsruhe, Germany) and dissolved in 20% methanol (Merck, Darmstadt, Germany) after 3 days of incubation. The neutralization titer was defined as the reciprocal of the highest serum dilution at which none of the triplicates showed signs of CPEs.

### 2.5. ELISpot Assay for SARS-CoV-2 S and M Proteins

SARS-CoV-2 specific T-cell responses were determined by interferon gamma (IFN-γ) enzyme-linked immunospot (ELISpot) assay using synthetic peptide pools of the SARS-CoV-2 spike (S1/S2) and membrane (M) protein (600 pmol/mL of each peptide; PepTivator^®^, Miltenyi Biotec, Bergisch Gladbach, Germany). The peptide pools mainly consisted of 15-mer sequences with 11 overlapping amino acids. In total, 250,000 mononuclear cells isolated from patient blood were incubated with the respective peptides and added onto anti-interferon-gamma-coated strip assay plates (Merck Millipore Ltd., Tullagreen, Ireland). After 19 h of incubation, substrate solution (Oxford Immunotec, Oxford, UK) was added and spots were analyzed by an ELISpot reader (AID Fluorospot, Autoimmun Diagnostika GmbH, Strassberg, Germany). Mean values of duplicate samples were considered and a total of at least three spots after subtraction of unstimulated (background) spots (spot increment) was defined as positive [17].

### 2.6. SARS-CoV-2 Specific Antigen Test

The quantification of SARS-CoV-2 nucleocapsid (N) antigen protein was performed with the same nasopharyngeal swabs as used for PCR testing and viral culture. SARS-CoV-2 N antigen was detected using the CLIA-based LIAISON^®^ SARS-CoV-2 antigen assay (DiaSorin, Saluggia, Italy) according to the manufacturer’s instructions. The assay has an analytical sensitivity of 22.0 TCID_50_/_mL_ [18,19].

### 2.7. Flow Cytometry

To assess T-cell, B-cell, and Natural Killer (NK) cell counts, a flow cytometry characterization was performed. The samples were hemolyzed and stabilized accordingly (Coulter Immunoprep, Beckman Coulter, Brea, CA, USA). Monoclonal antibodies were each conjugated to one of the following monochromes: PC5 (R Phycoerythrincyanin 5.1), Phycoerithrin (PE), Phycoerythrin—Texas Red-X (ECD) or Fluorescein isothiocyanate (FITC). The cells were stained for CD3 (mouse IgG1, PC5), CD4 (mouse IgG1, PE), CD19 (mouse IgG1, ECD), CD16 (mouse IgG1, PE) and CD56 (mouse IgG1, PE) surface proteins. Fluorescent microbeads (Flow-Count Fluorospheres, Beckman Coulter) were added to determine cell counts on a flow cytometer (Cytomitcs FC 500, Beckman Coulter).

### 2.8. SARS-CoV-2 PCR

Two different SARS-CoV-2 RT-PCR kits were used. The RealStar^®^ SARS-CoV-2 RT-PCR kit (Altona Diagnostics, Hamburg, Germany) with a detection limit of 625 copies/mL which targets S (spike) and E (envelope) genes of SARS-CoV-2. The Alinity m SARS-CoV-2 Assay (Abbott Laboratories, Chicago, IL, USA), which targets RNA-dependent RNA polymerase (RdRp) and nucleocapsid (N) genes, with a detection limit of 50 copies/mL, was also used. Samples in which SARS-CoV-2 could not be detected up to a cycle threshold (ct) of 43 were considered negative. Both tests were performed according to manufacturer’s instructions [19,20].

### 2.9. Statistics and Data Analysis

Statistical analyses were performed with IBM SPSS-Statistic 27 (SPSS Inc., Chicago, IL, USA) and GraphPad Prism 8 (GraphPad Software, San Diego, CA, USA) software. Datasets were analyzed with the independent *t*-test, Mann–Whitney test, Brown–Forsythe test, Welch ANOVA, Kruskal–Wallis test, Kaplan–Meier estimator and Spearman’s rank correlation coefficient. Univariate linear regression models were used to estimate relationships between the independent variables. *p* values of ≤0.05 were marked as statistically significant with ”*”, *p* values of ≤0.01 were marked with “**” and *p* values of ≤0.001 were marked with “***”. Correlation analyses were run for CD19^+^ B-cell numbers, SARS-CoV-2 neutralizing antibody titers, SARS-CoV-2 IgG ELISA antibody titers and IFNγ ELISpot increment for the spike protein(S1/S2), independent of whether patients had diabetes mellitus or an immunocompromisation.

## 3. Results

**Figure 1:**
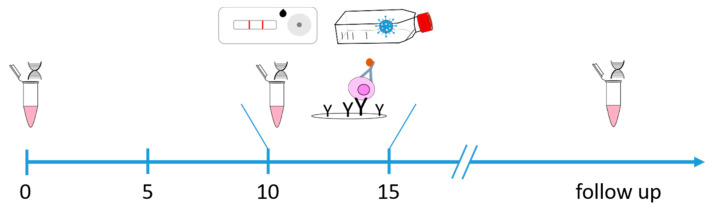
Study design. 79 patients were enrolled in the study; all of them had positive SARS-CoV-2 RT-PCR and were unvaccinated against SARS-CoV-2. Blood was drawn and nasopharyngeal swabs were taken approximately 10–15 days later to perform an antigen test, virus cultivation, an ELISpot assay and SARS-CoV-2 RT-PCR. Given the fact that the second SARS-CoV-2 PCR was positive, a follow-up PCR was performed around 7 days later.

Among others, age, immunodeficiency, and pre-existing diseases such as diabetes mellitus are known risk factors for a severe course of COVID-19. Accordingly, the clinical management of these vulnerable patient groups is challenging in terms of deisolation and transfer to outpatient medical care to warrant optimal primary healthcare. To understand the immunologic and virologic status (viral shedding) of these patients, we determined the humoral and cellular immune response of 79 hospitalized elderly ≥60 years old (*n* = 20), immunocompromised (*n* = 36) and diabetic (*n* = 23) unvaccinated COVID-19 patients and monitored them for SARS-CoV-2 RNA and virus shedding for up to 90 days upon hospitalization. SARS-CoV-2 RNA and viral loads were determined immediately after hospitalization, around days 10–15, and during follow-up (Figure 1). Blood samples for immunomonitoring and assessment of cellular and humoral immune responses were acquired at about 10–15 days after the first positive SARS-CoV-2-PCR result. If still hospitalized and with persistently positive SARS-CoV-2 RNA following the second PCR test, SARS-CoV-2 RNA was quantified again one week later. Figure 1 illustrates an overview of the study design.

### 3.1. Clinical Data

The characteristics of the patient cohorts investigated in the present study are summarized in Table 1. According to the risk factors for prolonged viral shedding and severe COVID-19, patients were divided into three subgroups: elderly patients (60 years or older; 60+), diabetes mellitus patients (DM) and immunocompromised patients (IM).

In total, 20 patients were over 60 years old and did not have immunodeficiency or diabetes mellitus. Thirty-six patients were immunocompromised (12 patients with malignant diseases, 8 solid organ transplant recipients (SOT), 4 patients with liver cirrhosis, 3 patients with permanent kidney replacement therapy, 3 patients with end-stage heart failure, 2 pregnant patients, 1 SOT and permanent kidney replacement therapy, 1 patient with Crohn’s disease, 1 bone marrow transplant recipient, 1 patient with chronic kidney disease). In this group, eight patients had a history of diabetes mellitus. Twenty-three patients suffered from diabetes mellitus without being immunocompromised.

There was a significant difference between the three patient groups regarding age (*p* = 0.03), severity of COVID-19 (*p* = 0.003), presence of the concomitant diseases hypertension (*p* = 0.02) and obesity (*p* = 0.03), therapy with Remdesivir (*p* = 0.004), and the number of nosocomial infections (*p* = 0.02; Table 1). Patients in the immunocompromised group were significantly younger than those in the 60+ group and were significantly less likely to suffer from hypertension than the diabetes mellitus group.

Importantly, immunocompromised patients had a significantly higher mortality and risk of severe COVID-19 when compared to elderly or diabetic patients. There were significantly more cases of severe COVID-19 among diabetes mellitus patients when compared to the elderly patients. COVID-19 treatment was conducted in accordance with international and national guidelines. In COVID-19 with low-dose oxygen requirements, antiviral therapy with Remdesivir was administered and, in case of further respiratory deterioration or in the later COVID-19 course, with Dexamethasone. With respect to therapy, there were significant differences between the patient groups. Remdesivir was used significantly less frequently in immunocompromised patients despite the often severe COVID-19 course due to impaired renal or hepatic function. Instead, therapy with Dexamethasone was conducted when necessary. Notably, immunocompromised patients showed significant higher rates of nosocomial infections than elderly and diabetes mellitus patients. In total, 13 of 20 patients with a nosocomial infection were in the immunocompromised group. Beyond that, no significant clinical differences could be detected between the three groups.

### 3.2. Immunomonitoring

#### 3.2.1. Cellular Immune Status

**Figure 2:**
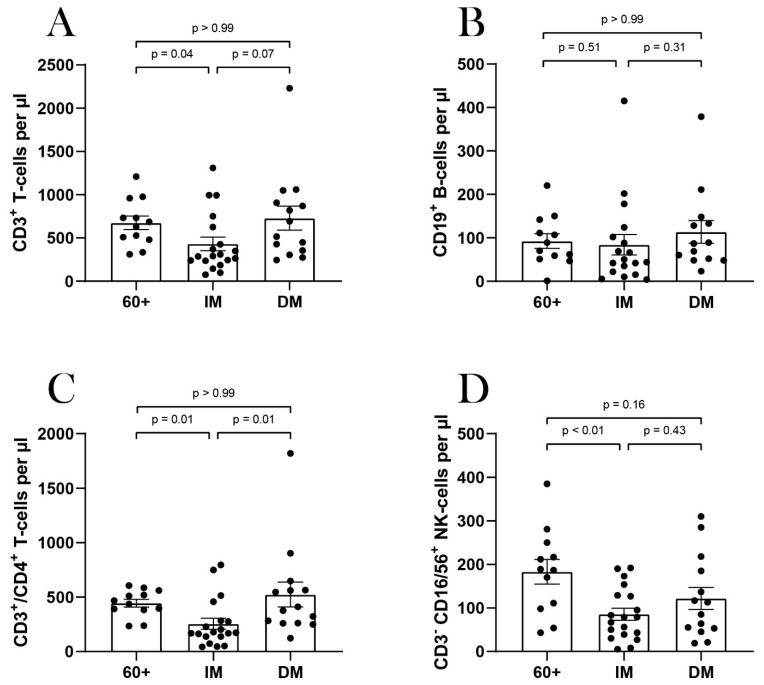
Cellular immune status of elderly (60+), immunocompromised (IM) and diabetes mellitus (DM) patients: Total numbers of CD3^+^ cells (**A**); CD19^+^ B-cells (**B**), CD3^−^, CD16/56^+^ cells (**C**) and CD3^+^, CD4^+^ T-cells (**D**) were determined for 45 patients. Each dot (●) represents one patient. A comparison between the groups was performed with the Mann–Whitney test. Statistical significance was set at the level of *p* < 0.05.

The cellular immune status was examined in 45 patients during hospitalization (Figure 2). Of these, 12 patients were elderly patients (60+), 19 patients were immunocompromised, and 14 patients suffered from diabetes mellitus without being additionally immunocompromised. Immunocompromised patients had significantly fewer CD3^+^ T-cells and CD3^+^CD4^+^ T-cells compared to elderly patients. Immunocompromised patients also had significantly fewer CD3^+^CD4^+^ T-cells than diabetes mellitus patients and fewer CD3^−^, CD16/56^+^ natural killer (NK) cells compared to elderly patients. No significant differences in terms of CD19^+^ B-cells were detected between the three subgroups (Figure 2).

#### 3.2.2. Cellular Immunity

To determine the extent of SARS-CoV-2-specific T-cell responses to SARS-CoV-2 spike (S1/S2) and membrane proteins (M), IFNγ-ELISpot analyses were performed from 51 blood samples. Therefore, peripheral blood mononuclear cells (PBMCs) isolated from 12 elderly patients, 25 immunocompromised patients, and 14 diabetes mellitus patients were stimulated with peptide pools of the SARS-CoV-2 spike (S1/S2) or membrane (M) protein. Subsequently, the cellular immune response was quantified by assessing the number of IFNγ-positive spots. T-cell responses of elderly, immunocompromised and diabetes mellitus patients were compared with the T-cell responses of a convalescent group. The convalescent group consisted of 20 younger convalescents with a mean age of 41 years (range 19 + 58 years) and a mean time of sampling after PCR-confirmed SARS-CoV-2 infection of 64 days (range 24–84 days). None of these convalescents were hospitalized due to SARS-CoV-2. In total, 40 patients showed a T-cell response to the S1/S2 peptide pool and 44 patients showed a T-cell response to the M peptide pool in the SARS-CoV-2-IFNγ-ELISpot assay. There was no detectable S1/S2 and M protein specific cellular immune response in five patients with immunosuppression.

**Figure 3:**
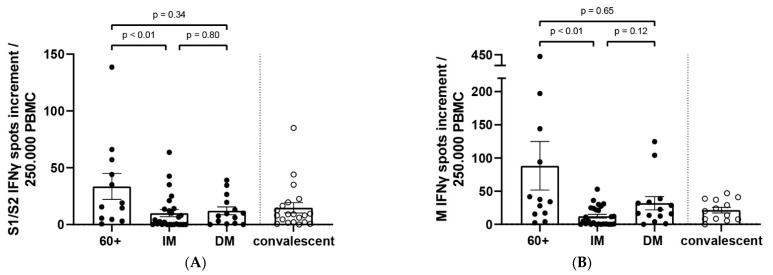
T-cell responses of elderly (60+), immunocompromised (IM) and diabetes mellitus (DM) patients compared to a convalescent cohort: S1/S2-protein-specific T-cell responses (**A**) and M protein specific T-cell responses (**B**) for 51 patients and 20 convalescent was determined via ELISpot assay. Each dot (●) represents one patient and each hollow dot (○) represents one convalescent. Comparison between the groups was performed with the Mann–Whitney test. Statistical significance was set at the level of *p* < 0.05.

SARS-CoV-2-specific T-cell responses were significantly lower in immunocompromised patients compared to elderly 60+ patients regarding both the S1/S2 and the M peptide pool. No significant difference could be found in the cellular immune response for the convalescent and 60+, IM or DM patients (Figure 3A,B).

#### 3.2.3. Humoral Immunity

The humoral immune response was assessed by determining SARS-CoV-2 IgG ELISA titers and neutralizing antibody titers. SARS-CoV-2 IgG ELISA titers were assessed in 70 out of 79 patients. A total of 9 out of 70 patients received anti-SARS-CoV-2 antibodies or convalescent plasma therapy and were excluded from this analysis. One patient received plasmapheresis due to concomitant disease before sampling and was also excluded. The SARS-CoV-2 IgG ELISA titers were determined in sera from 13 elderly (60+), 26 immunocompromised (IM), and 21 diabetes mellitus (DM) patients.

Overall, in 16 patients, no SARS-CoV-2 IgG ELISA antibody response was detected. Most of these patients were immunocompromised (*n* = 11), but 3 elderly and 2 diabetic patients also did not show an antibody response. The patients that did not show SARS-CoV-2-neutralizing antibodies also had no measurable cellular immune response.

**Figure 4:**
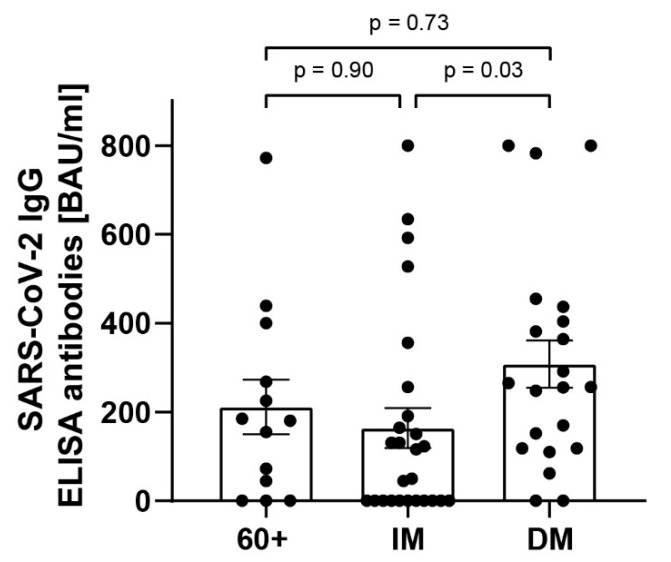
SARS-CoV-2 IgG ELISA levels of elderly (60+), immunocompromised (IM) and diabetes mellitus (DM) patients. SARS-CoV-2 IgG ELISA levels of 60 patients were determined via antibody assay. Each dot (●) represents one patient. Comparison between the groups was performed with the Kruskal–Wallis test. Statistical significance was set at the level of *p* < 0.05.

Thus, only about 73% of the total patient cohort (*n* = 44) had detectable SARS-CoV-2 IgG levels and immunocompromised patients showed significantly lower levels than diabetes mellitus patients. Figure 4 provides an overview of the SARS-CoV-2 IgG ELISA distribution in each group.

**Figure 5:**
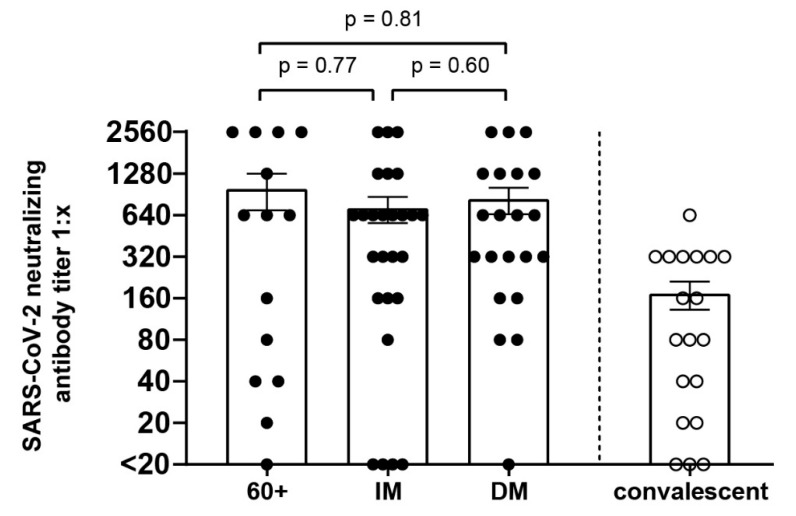
SARS-CoV-2-eutralizing antibody titers of elderly (60+), immunocompromised (IM) and diabetes mellitus (DM) patients compared with a group of convalescents: Virus neutralization assays were performed for 62 patients and 19 convalescent donors. Each dot (●) represents one patient. (○) represents one convalescent. Comparison between the groups was performed with the Mann–Whitney test. Statistical significance was set at the level of *p* < 0.05.

Neutralizing SARS-CoV-2 antibodies were evaluated in 71 out of 79 patients. A total of 15 patients were elderly, 33 were immunocompromised and 23 were diabetic patients. A total of 9 out of 71 patients received anti-SARS-CoV-2 antibodies or convalescent plasma therapy and were, therefore, excluded from this analysis. The reference group consisted of the same convalescent patients as the group for cellular immunity, excluding one patient that did not give a sample for the evaluation of neutralizing SARS-CoV-2 antibodies. A total of 13 elderly, 23 immunocompromised, 20 diabetic patients, and 16 convalescents had detectable SARS-CoV-2-neutralizing antibodies (Figure 5).

### 3.3. Correlation Analyses

**Figure 6:**
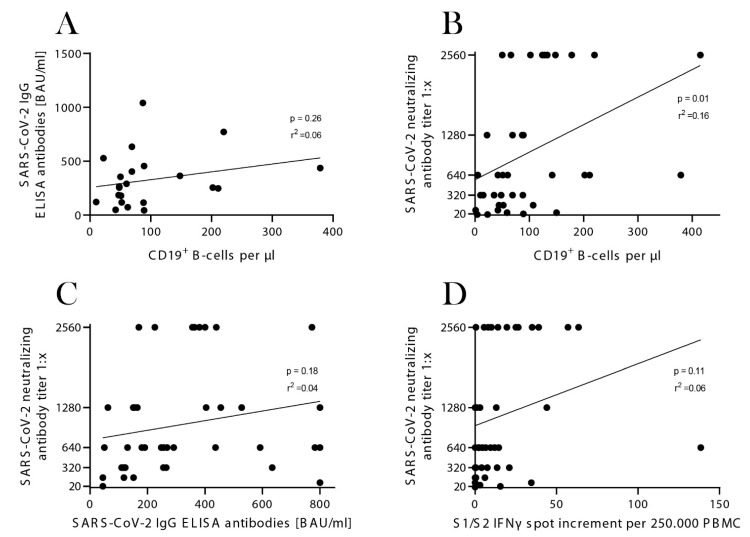
Correlation analyses for SARS-CoV-2 IgG ELISA antibody titers and CD19+ B-cell numbers (**A**), SARS-CoV-2 neutralizing antibody titers and CD19+ B-cell numbers (**B**), SARS-CoV-2 neutralizing antibody titers and SARS-CoV-2 IgG ELISA antibody titers (**C**) and SARS-CoV-2 neutralizing antibody titers and IFNγ ELISpot increment for the spike (S1/S2) protein (**D**). Each dot (●) represents one patient.

To better understand the associations between different aspects of the humoral and cellular immune response, correlation analyses were performed. Our data indicated a significant correlation between SARS-CoV-2-neutralizing antibody titers and CD19+ B-cell numbers (*p* = 0.01, r2 = 0.16, *n* = 39). There was no correlation between SARS-CoV-2 IgG ELISA antibody titers and CD19+ B-cell numbers (*p* = 0.26, r2 = 0.06, *n* = 22). There was also no significant correlation between SARS-CoV-2-neutralizing antibody titers and SARS-CoV-2 IgG ELISA antibody titers (*p* = 0.18, r2 = 0.04, *n* = 42) or IFNγ ELISpot increment (S1/S2) (*p* = 0.11, r2 = 0.06, *n* = 47; Figure 6A–D).

### 3.4. Nasopharyngeal Sampling

Nasopharyngeal swabs were taken about 10–15 days after the first positive SARS-CoV-2 PCR test. This swab was used to perform a SARS-CoV-2 antigen test, SARS-CoV-2 RT-PCR and virus cultivation to determine if replication-competent virus was still present in the elderly, immunocompromised and diabetes mellitus patients after symptomatic COVID-19 over an extended period.

A total of 64 samples were tested for SARS-CoV-2 antigen, and SARS-CoV-2 was detectable in 9 out of 29 immunocompromised patients. This could not be detected in the 17 swabs of elderly patients or in the 18 swabs of diabetic patients. All nine samples with detectable SARS-CoV-2 antigen had a ct-value of 25 or lower.

Nasopharyngeal swabs from 79 patients were tested via SARS-CoV-2 PCR. In total, 16/20 elderly patients (80%) showed SARS-CoV-2 RNA with a mean cycle threshold of 33. A total of 29/36 samples (80.65%) from immunocompromised patients were PCR-positive with a mean ct of 30 and 11/23 samples (47.83%) from diabetes mellitus patients were PCR-positive with a mean ct of 32. Most of these patients were deisolated and discharged from the hospital after consultation with the local health department and/or the Institute for Hospital Hygiene. However, 24 out of 56 patients showing positive PCR results were still hospitalized one week later. In a follow-up SARS-CoV-2 PCR testing, 3/5 elderly, 9/14 immunocompromised and 2/5 diabetes mellitus patients out of those 24 patients continued to be PCR-positive.

**Figure 7:**
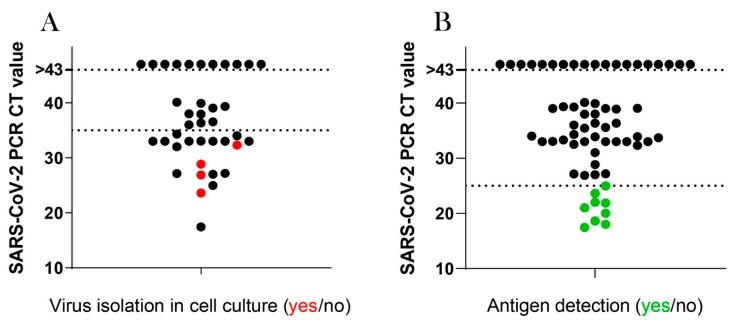
Correlation analyses for SARS-CoV-2 PCR ct-values and virus cultivation (A) or antigen detection (B). Nasopharyngeal swabs from 79 COVID-19 patients were tested for SARS-CoV-2 RNA via PCR. Additionally, 39 samples of patients with a positive SARS-CoV-2 PCR were inoculated on Vero E6 cells to detect infectious virus (**A**) and 64 samples were tested for SARS-CoV-2 N antigen (**B**). Red dots (●) in (**A**) indicate samples with cultivatable SARS-CoV-2 and green dots (●) in (**B**) indicate samples with positive SARS-CoV-2 antigen test.

A total number of 39 nasopharyngeal swabs were tested in cell culture for infectious virus. Nine of these nasopharyngeal swabs were obtained from elderly patients, 20 from immunocompromised and 10 from diabetes mellitus patients. Plaque formation was observed in four samples, all with a ct-value below 33. A positive SARS-CoV-2 viral culture was obtained in four patients (Figure 7). Three out of those four patients were immunocompromised and one was an elderly patient. Virus could be cultivated in 1 out of 20 samples (5%) with a ct-value of 30 to 43 and in 3 out of 8 samples (37.5%) with a ct-value below 30.

A total of 24 patients received a follow-up nasopharyngeal swab, which was conducted around 7 days after the first virus cultivation. In four of those swabs, SARS-CoV-2 was isolated via PCR, and in two of those swabs, SARS-CoV-2 was cultivatable in vitro.

### 3.5. Clinical Outcome

**Figure 8:**
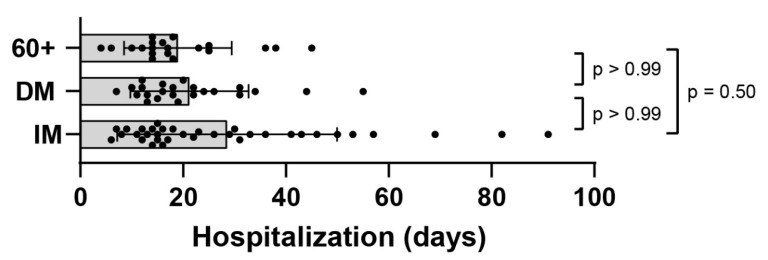
Duration of hospitalization of elderly (60+), immunocompromised (IM) and diabetes mellitus (DM) patients. Days in hospital for a cohort of 78 patients. Each dot (●) represents one patient. Brown–Forsythe test and Welch ANOVA were used as statistical tests. Statistical significance was set at *p* ≤ 0.05.

The duration of hospitalization for elderly, immunocompromised and diabetes mellitus patients was compared. Mean length of hospitalization was 24 days (range 4–91 days) for the total patient cohort. On average, elderly patients were hospitalized for 19 days (range 4–45 d), immunocompromised patients for 28.6 days (range 9–91 d) and diabetes mellitus patients for 21.3 days (range 7–55 d) (Figure 8). Of note, some immunocompromised patients were hospitalized for more than 50 days; however, the groups did not differ significantly.

### 3.6. Overall Survival

**Figure 9:**
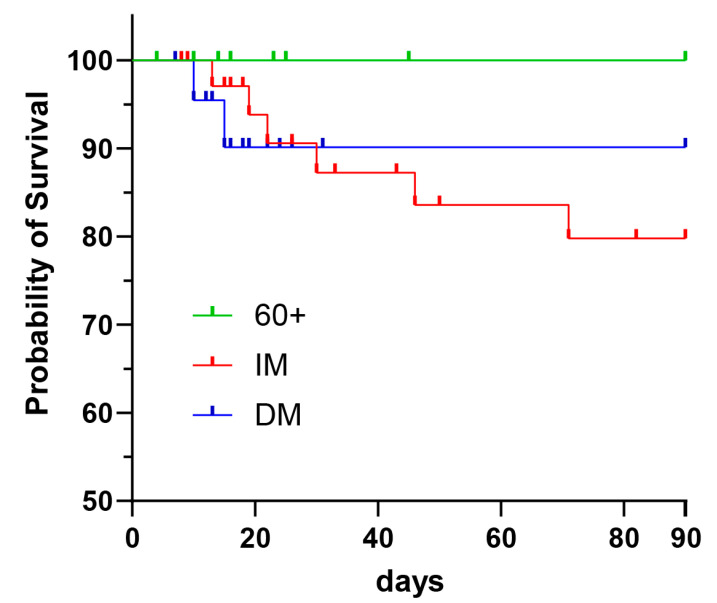
Kaplan–Meier estimate showing the probability of survival for elderly (60+), immunocompromised (IM) and diabetes mellitus (DM) patients. Patients’ survival was monitored for 90 days after the first positive SARS-CoV-2 PCR.

Next, we examined the differences in the survival probability of our study cohort over a follow-up period of 90-days. Importantly, immunocompromised patients showed a lower probability of survival (75%) compared to diabetes mellitus patients (80%) or elderly patients (100%). However, the difference was not significant (Figure 9).

## 4. Discussion

In the present study, we evaluated the humoral and cellular immunity, overall survival and infectivity in a cohort of 79 unvaccinated, hospitalized COVID-19 patients. The study cohort comprised patients with high risk factors predisposed to severe COVID-19, such as advanced age, immunocompromisation and diabetes mellitus. Our data suggest that immunocompromised patients have a weaker cellular immune response, while the humoral immune response seems to be decent. Immunosuppressed patients seem to shed virus over a prolonged period.

The combined results of our study not only characterize the immune response but should further have a meaningful impact on the in-hospital management of high-risk COVID-19 patients, which is often as delicate as it is important.

Regarding the humoral immune response to SARS-CoV-2 infection, it is still unclear if pre-existing comorbidities (especially great age and immunocompromisation) are associated with lower antibody levels [21]. All patients examined in our study had a moderate to severe COVID-19 course with hospitalization and, in some cases, oxygen support, whereas none of the convalescents were hospitalized.

We compared SARS-CoV-2-neutralizing antibody titers of the study cohort with those of convalescent patients recovered from a mild or asymptomatic SARS-CoV-2 infection without relevant comorbidities. Of note, convalescent group samples were obtained after SARS-CoV-2 infection. The neutralizing antibody titers of the convalescents did not significantly differ from those of the elderly, immunocompromised or diabetes mellitus patients. However, the heterogeneity in terms of infection severity and sample period could have masked a difference in neutralizing antibody titers. Although SARS-CoV-2-neutralizing antibodies remain detectable in the first few months after mild COVID-19 [22,23], their levels may decrease with time after SARS-CoV-2 infection.

In addition, it has been reported that the severity of COVID-19 affects antibody levels, e.g., milder COVID-19 symptoms lead to lower antibody levels [24,25]. According to these data, we identified significantly reduced SARS-CoV-2 IgG ELISA antibody levels in immunocompromised patients compared to diabetes mellitus patients, but not compared to elderly patients. The elderly patients in our cohort showed significantly more moderate COVID-19 cases than the diabetes mellitus patients, possibly leading to lower SARS-CoV-2 antibody titers. This evidences a lower humoral immune response in immunocompromised patients. Of note, one-third of the immunocompromised patients in our cohort did not develop any humoral immune response during the acute COVID-19 phase. This is consistent with recent studies showing that immunocompromised patients are significantly more likely to remain seronegative after COVID-19 [26,27,28].

Likewise, significantly lower T-cell-specific immunity was observed in immunocompromised patients compared to elderly patients. This is in accordance with recently published data showing a significantly reduced T-cell response in solid organ transplant recipients (SOT) in the acute phase of COVID-19 [29]. Overall, the cellular immune response after COVID-19 is not well characterized in immunocompromised patients. Of note, immunocompromised patients that did not develop measurable SARS-CoV-2-neutralizing antibodies also had no measurable T-cell responses. Significantly lower T-cell-specific immune response in immunocompromised patients after SARS-CoV-2 vaccination could support the hypothesis of a reduced response [15,30,31,32]. However, recent studies also demonstrated no difference between the T-cell-specific immune response of immunocompromised and immunocompetent patients [33,34,35]. Therefore, further research is needed to define possible differences in T-cell-specific immunity in immunocompromised and immunocompetent patients.

Patients with diabetes mellitus not only had more severe COVID-19 compared with geriatric patients, but, similarly to immunocompromised patients, had a lower T-cell-specific immune response, although this did not reach the significance level. This is consistent with recent evidence suggesting that impaired T-cell responses attributable to multifactorial factors may predispose diabetic patients to a more severe COVID-19 course [36].

Our correlation analyses revealed that higher CD19^+^ B-cell numbers were associated with higher SARS-CoV-2-neutralizing antibody titers. This is consistent with a prior study showing that patients barely develop antibody responses if they have a deficiency of B-cells [37]. No correlation was observed between SARS-CoV-2 IgG ELISA antibody titers and neutralizing antibody titers, which is contrary to the findings of others [37,38]. Furthermore, there was no correlation between SARS-CoV-2-neutralizing antibody titers and IFNγ ELISpot increment. These findings are in line with prior studies [26]. However, the correlation analyses are limited due to the low number of samples.

In line with our findings showing no deficiency in humoral or cellular immune response in elderly and diabetic SARS-CoV-2-positive patients, infectious virus was isolated in only one case. Of note, in samples from the immunocompromised patients, infectious virus was isolated in only three cases. Thus, our results indicate that elderly, immunocompromised and diabetic patients are not susceptible to prolonged virus shedding. This is in line with recent data demonstrating that the median duration of infectious-virus excretion was 8 days after the onset of symptoms, and excretion rates reached lower than 5% after 15.2 days [39]. Other study groups were unable to culture virus at all 8 days after the onset of symptoms [40]. Other authors suggest that persistent viral RNA shedding might be associated with different factors, such as receiving glucocorticoid treatment or suffering from severe COVID-19 [13,39]. One third of our patients—most of them immunocompromised or diabetic—received dexamethasone therapy. However, we cannot exclude that the prolonged detection of SARS-CoV-2 RNA was not caused solely by the underlying comorbidities, but also by the glucocorticoid therapy those patients received.

In previous studies, vital virus could not be cultured when ct-values were above 24 [41]. In contrast to those findings, we were able to isolate virus in samples with a ct-value of 27 (from an elderly patient) and in samples with a ct-value of 24, 33 and 29 (from immunocompromised patients). The elderly, immunocompromised and diabetic patients in our study were identified as PCR-positive for a longer period than reported in recent studies. The mean time from symptom onset to the first negative RT-PCR result reported in the literature was 9.5 (6.0–11.0) days [42]. In our study population, mean time to SARS-CoV-2 PCR control was 16 days. These findings are in line with recently published data showing prolonged virus-shedding in patients with chronic kidney disease or tumor patients [43,44]. At that time most patients remained persistently PCR-positive for SARS-CoV-2, and thus had to be isolated according to the Robert Koch Institute (RKI) recommendations at that time. The consequence of the strict isolation measures was a prolonged hospitalization for the patients in our cohort (24 days) compared to the average duration for inpatients with COVID-19 in Germany (14.3 days) [45]. Of importance, despite persistent SARS-CoV-2 PCR detection, only low levels of replication-competent virus could be cultured in our cohort.

Those elongated hospitalization times do not only burden the medical system with high numbers of avoidable inpatients but could additionally endanger patients. Our clinical data included information about the frequency of antibiotic therapy and the necessity of this therapy for a nosocomial infection. The analysis displayed that 36% of immunocompromised patients suffered from a nosocomial infection (13 of 36 patients). This number stands in contrast to the average percentage of nosocomial infections in Germany for the year 2020 (6.8%). Diabetic (23%) and elderly patients (10%) also showed a higher number of nosocomial infections, although the rate not as high as for the immunosuppressed patients. Before the COVID-19 pandemic, only 5.6% of all hospital patients had a nosocomial infection [46]. The authors suggested an increase in infections during the pandemic due to a higher number of patients being treated per nurse and the lack of protective gear. However, an elongated hospitalization time due to unconclusive isolation requirements can also represent a hazard, especially as patients with imprecise isolation requirements also have a higher risk of nosocomial infections, namely, immunocompromised patients [46]. Nosocomial infections are associated with higher mortality. In addition, this leads to rising economical costs.

However, in our study, the mortality in our subgroups was below the average at that time in Germany [45]. The experience of all our medical staff ward members with high-risk patients’ treatment might be a possible explanation for this.

It is key to discharge patients right after appropriate treatment has been given to decrease the risk of developing nosocomial infections and optimize the usage of medical health care system capacity. To date, most national and international recommendations advocate extending isolation and precautions for severely ill COVID-19 or severely immunocompromised persons to up to 20 days after the onset of symptoms and concurrent improvement in other symptoms. No clear procedural evidence-based guidance on handling at-risk individuals with persistent RNA detection is available at present. Recent work has suggested that seroconversion could be used as an additional parameter for deciding when to end isolation regimens [42]. Other recent studies even suggest non-infectivity in PCR-positive patients with a neutralizing antibody titer above 1:20. Thus, independent predictors for the detection of infectious SARS-CoV-2 from the respiratory tract were high viral loads and an absence of neutralizing antibodies in serum, but not immunodeficiency [39]. Most of our patients, had a neutralizing antibody titer well above 1:50, despite a positive SARS-CoV-2 PCR result. Since the presence of infectious virus could not be detected in most cases, further quarantine measures probably could have been omitted.

Our study has some limitations. Firstly, it is a monocentric study, only considering patients from one university hospital. Secondly, the patients in the immunocompromised cohort are quite heterogeneous with respect to their underlying disease. Our data indicate that this heterogenous group seems to have comparable immune responses. Neither age, concomitant diabetes nor subgroups within immunocompromised patients indicated strong deviations in the immune response or viral shedding. Our data also show no association between the kind of treatment for COVID-19 and the occurrence of nosocomial infection or virus shedding. Our results are limited due to the number of patients in these subgroup, and further studies are required. Thirdly, our prospective study includes immunocompromised patients, elderly patients and patients with diabetes mellitus, but not a younger control group. Comparing the immune status of our study cohort with a younger control group may reveal possible age-dependent differences in SARS-CoV-2-specific humoral and cellular immunity.

In conclusion, our data indicate significantly lower SARS-CoV-2 IgG ELISA antibodies in immunocompromised patients compared to diabetic patients, and a lower T-cell-specific immune response compared to geriatric patients. Infectious virus was only isolated from four samples with a cycle threshold of 33 or lower. Furthermore, no difference could be found in the magnitude of SARS-CoV-2-neutralizing antibody levels in the immunocompromised patients who developed antibody titers compared to elderly and diabetes mellitus patients. Therefore, the earlier discharge of high-risk patients (including immunocompromised patients) after the appearance of antibodies could be a way to stratify isolation measures. However, additional studies are needed to confirm these findings in larger cohorts.

## Figures and Tables

**Table 1:** Characteristics of the total patient cohort with three subgroups: elderly (60+), immunocompromised (IM) and diabetes (DM) patients. Patients were classified according to the COVID-19 category given by the European Centre for Disease Prevention and Control (ECDC) classification among all cases: hospitalization (a), severe hospitalization (b), and death (c). Comparison between sex, age, length of hospital stay in days, time of sampling, ECDC criteria, hypertension, COPD, obesity, artificial ventilation, antibody/plasma therapy, Remdesivir and dexamethason therapy of the patients was performed with an independent *t*-test. Statistical significance was set at the level of *p* < 0.05.

	Total	60+	IM	DM	*p* Value 60+/IM	*p* Value 60+/DM	*p* Value IM/DM
Number, *n*	79	20	36	23			
Sex, men/women	49/30	9/11	24/12	16/7	0.11	0.1	0.82
Age, y ± SD	67.7 ± 14.5	73.4 ± 10.1	63.3 ± 17.7	69.6 ± 9.9	0.03	0.66	0.22
Length of hospital stay, d (range)	24 (4–91)	19 (4–45)	28.6 (6–91)	21.3 (7–55)	0.38	>1	>1
Time of sampling, d ± SD	16 ± 7.7	14.4 ± 8.2	15.8 ± 7.5	17.8 ± 7.4	0.70	0.10	0.72
ECDC classification, *n*	23 a, 51 b, 5 c	9 a, 11 b, 0 c	13 a, 18 b, 5 c	1 a, 22 b, 0 c	0.2	0.003	0.15
Hypertension, *n* (%)	49 (62)	11 (55)	19 (53)	19 (83)	0.87	0.05	0.02
COPD, *n* (%)	8 (10)	2 (10)	4 (11)	2 (9)	0.9	0.88	0.76
Obesity, *n* (%)	27 (34)	6 (30)	9 (25)	12 (52)	0.64	0.14	0.03
Artificial ventilation, *n* (%)	11 (14)	1 (5)	6 (17)	4 (17)	0.21	0.21	0.94
Antibody/plasma therapy (%)	12 (15)	2 (10)	8 (22)	2 (9)	0.25	0.88	0.18
Remdesivir, *n* (%)	25 (32)	9 (45)	5 (14)	11 (48)	0.01	0.85	0.004
Dexamethason, *n* (%)	26 (33)	5 (25)	11 (31)	10 (43)	0.66	0.2	0.31
Antibiotic therapy, *n* (%)	50 (63)	10 (50)	24 (67)	16 (70)	0.23	0.2	0.82
Nosocomial infektion, *n* (%)	20 (25)	2 (10)	13 (36)	5 (23)	0.02	0.31	0.21

## Data Availability

The raw data supporting the conclusions of this article will be made available by the authors upon request.
